# The forkhead box C1 (FOXC1) transcription factor is downregulated in acute promyelocytic leukemia

**DOI:** 10.18632/oncotarget.21101

**Published:** 2017-09-20

**Authors:** Emiliano Fabiani, Giulia Falconi, Nélida Inés Noguera, Ernestina Saulle, Laura Cicconi, Mariadomenica Divona, Cristina Banella, Alessandra Picardi, Anna Maria Cerio, Letizia Boe, Massimo Sanchez, Elvira Pelosi, Ugo Testa, Francesco Lo-Coco, Maria Teresa Voso

**Affiliations:** ^1^ Università di Roma Tor Vergata, Dipartimento di Biomedicina e Prevenzione, Rome, Italy; ^2^ Fondazione Santa Lucia, Laboratorio di Neuro-Oncoematologia, Rome, Italy; ^3^ Istituto Superiore di Sanità, Centro Nazionale per la Ricerca e la Valutazione Preclinica e Clinica dei Farmaci, Rome, Italy; ^4^ Istituto Superiore di Sanità, Dipartimento di Ematologia ed Oncologia, Rome, Italy; ^5^ Istituto Superiore di Sanità, Grandi Strumentazioni e Core Facilities, Rome, Italy

**Keywords:** acute promyelocytic leukemia, forkhead box C1, ATRA, decitabine, epigenetic regulation

## Abstract

Forkhead box (FOX) genes encode transcription factors, which regulate embryogenesis and play an important role in hematopoietic differentiation and in mesenchymal niche maintenance. Overexpression of the family member FOXC1 has been reported in solid tumors and acute myeloid leukemia (AML).

We studied FOXC1 expression and function in acute promyelocytic leukemia (APL) and normal hematopoietic progenitors. FOXC1 mRNA and protein levels were significantly lower in primary marrow samples from 27 APL patients, as compared to samples obtained from 27 patients with other AML subtypes, and 5 normal CD34+ hematopoietic cells. FOXC1 expression significantly increased in APL samples at the time of remission following consolidation treatment. In cell lines overexpressing PML-RARA, and in the NB4 t(15;17)-positive cell line, FOXC1 expression was lower than in other non-APL cell lines, and increased following treatment with all-trans retinoic acid (ATRA), due to functional binding of ATRA to the FOXC1 promoter region. Reduced FOXC1 expression was also associated to DNA hypermethylation of the +354 to +568 FOXC1 region, both in primary APL, and in NB4 cells. Treatment of NB4 cells with decitabine demethylated FOXC1 and upregulated its expression.

Our findings indicate that FOXC1 is consistently repressed in APL due to hypermethylation and the presence of the PML-RARA rearrangement. A potential role of hypomethylating treatment in advanced APL remains to be established.

## INTRODUCTION

Acute promyelocytic leukemia (APL) is a well-defined type of acute myeloid leukemia (AML), characterized by the balanced t(15,17) chromosomal translocation, resulting in the fusion of the promyelocytic leukemia (PML) and retinoic acid receptor-α (RARA) genes. The aberrant oncogenic protein PML-RARA blocks myeloid differentiation at the promyelocyte stage by exerting dominant negative effects on wild-type RARA and PML genes, through transcriptional and post-transcriptional mechanisms. In particular, PML-RARA constitutively represses RA-target genes by recruiting chromatin remodelling proteins such as DNA methyltransferases, histone methyltransferases and methyl-binding domain proteins [1]. The differentiation block is further accomplished by binding to RA-response elements in the promoter region of myeloid transcription factor PU.1, the tumor suppressor PTEN, and stimulating transcription of pro-leukemogenic genes driven by hypoxia-induced factors (HIFs) [2–4]. Disruption of nuclear bodies, and blunting of the p53 response to DNA damage through p53 deacetylation have been reported as further mechanisms of cell immortalization [5].

Forkhead box (FOX) genes encode transcriptional factors which act as regulators of embryogenesis, and are involved in fundamental processes of cell differentiation in adults [6–7]. FOX genes are deregulated in solid tumors and their overexpression has been correlated with poor differentiation and unfavourable prognosis [8–11]. In particular, in melanoma cells and tissues, the promoter region of the family member FOXC1 is hypomethylated compared to normal tissues and this leads to upregulation of its expression [12]. Accordingly, hypomethylating treatment of the M219 and M15 melanoma cell lines upregulated FOXC1 protein expression [12].

Overexpression of FOXC1 has been reported in nearly 20% of primary AML samples, where FOXC1 has been shown to be involved in the monocyte/macrophage differentiation block and in the increased clonogenic potential of AML cells [13–14]. In the normal hematopoietic system, FOXC1 is essential for development and maintenance of the mesenchymal niche for stem and progenitor cells [15]. The role of FOX genes in APL is currently unknown.

We analysed FOXC1 gene expression and protein levels in APL primary samples, as compared to other AML subtypes and normal hematopoietic cells. In addition, we investigated, using cell line models, the mechanisms of FOXC1 deregulation in APL.

## RESULTS

### Characterization of FOXC1 expression in APL and other AML subtypes

**Figure 1:**
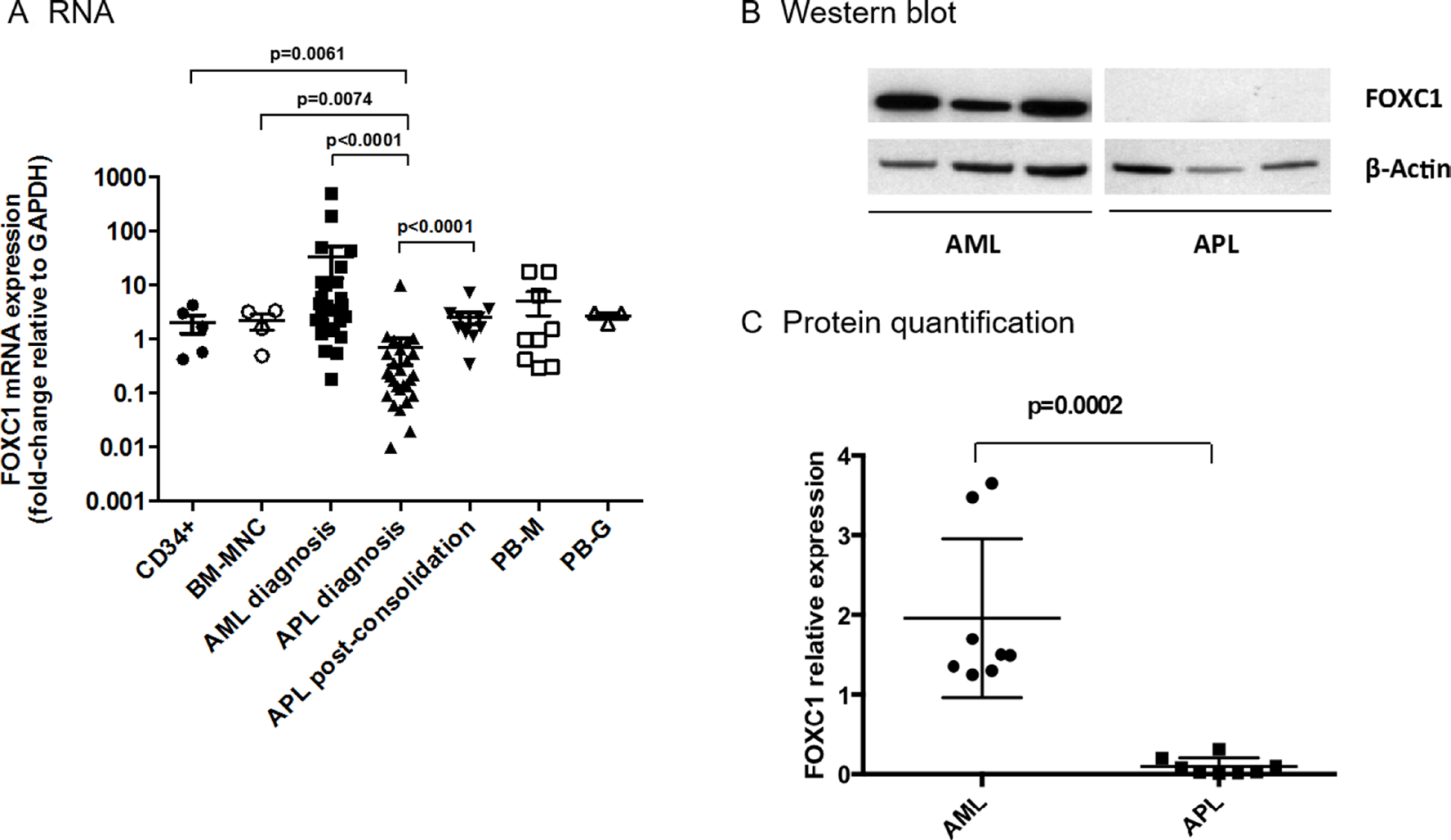
FOXC1 expression in AML, APL and controls (**A**) Expression of FOXC1 mRNA was studied in CD34+ cells isolated from cord blood (*n* = 2) and BM (*n* = 3), in normal BM-MNC (*n* = 4), in the BM-MNC of patients with AML (*n* = 27), or APL (*n* = 27), collected at the time of initial diagnosis, and of APL patients (*n* = 11), at the post-consolidation phase. Mature PB-monocytes (M) and granulocytes (G) served as further controls. (**B**) Western blot showing FOXC1 and β-Actin protein expression in six representative primary AML and APL samples collected at the time of initial diagnosis (western blot images are assembled for figure usability in respect of original image acquisition and no specific features are obscured, moved, removed, or introduced). (**C**) Quantification of FOXC1 protein expression in BM-MNC samples from patients with AML (*n* = 8), or APL (*n* = 8), at the time of initial diagnosis.

FOXC1 RNA expression was significantly downregulated in APL, compared to other AML subtypes (*n* = 27 APL vs 27 AML, *p* = 0.0001, Figure 1A). These data were confirmed by revising the published TCGA data set (http://cancergenome.nih.gov/dataportal/data/about), where FOXC1 expression in APL was 10-fold lower than that of other AML samples (Supplementary Figure 1). In APL, MNC from patients with a high-risk disease, classified according to the "Sanz score", tended to express FOXC1 at levels lower than those from patients with a low-intermediate risk APL, though the low number of high-risk APL tested might be too low to draw any conclusions (data not shown) [16]. Normal CD34+ hematopoietic cells isolated from cord blood (*n* = 2) or BM (*n* = 3) expressed FOXC1 at levels similar to those observed in AML, but significantly higher than those detected in APL (*p* = 0.0061, Figure 1A). FOXC1 expression significantly increased in the BM-MNC of patients with APL, collected after consolidation treatment according to the AIDA2000 or APL0406 protocols (*p* < 0.0001, Figure 1A) [17–18]. FOXC1 expression was similar in all other cell subsets, including normal BM-MNC, mature PB-monocytes and granulocytes (Figure 1A).

Down-regulation of FOXC1 in APL was confirmed at the protein level. Using western blot, we found that FOXC1 protein expression was very low/undetectable in 8 APL samples when compared to 8 non-APL AMLs (*p* = 0.0002, Figure 1B and 1C).

### FOXC1 methylation in AML and APL

**Figure 2:**
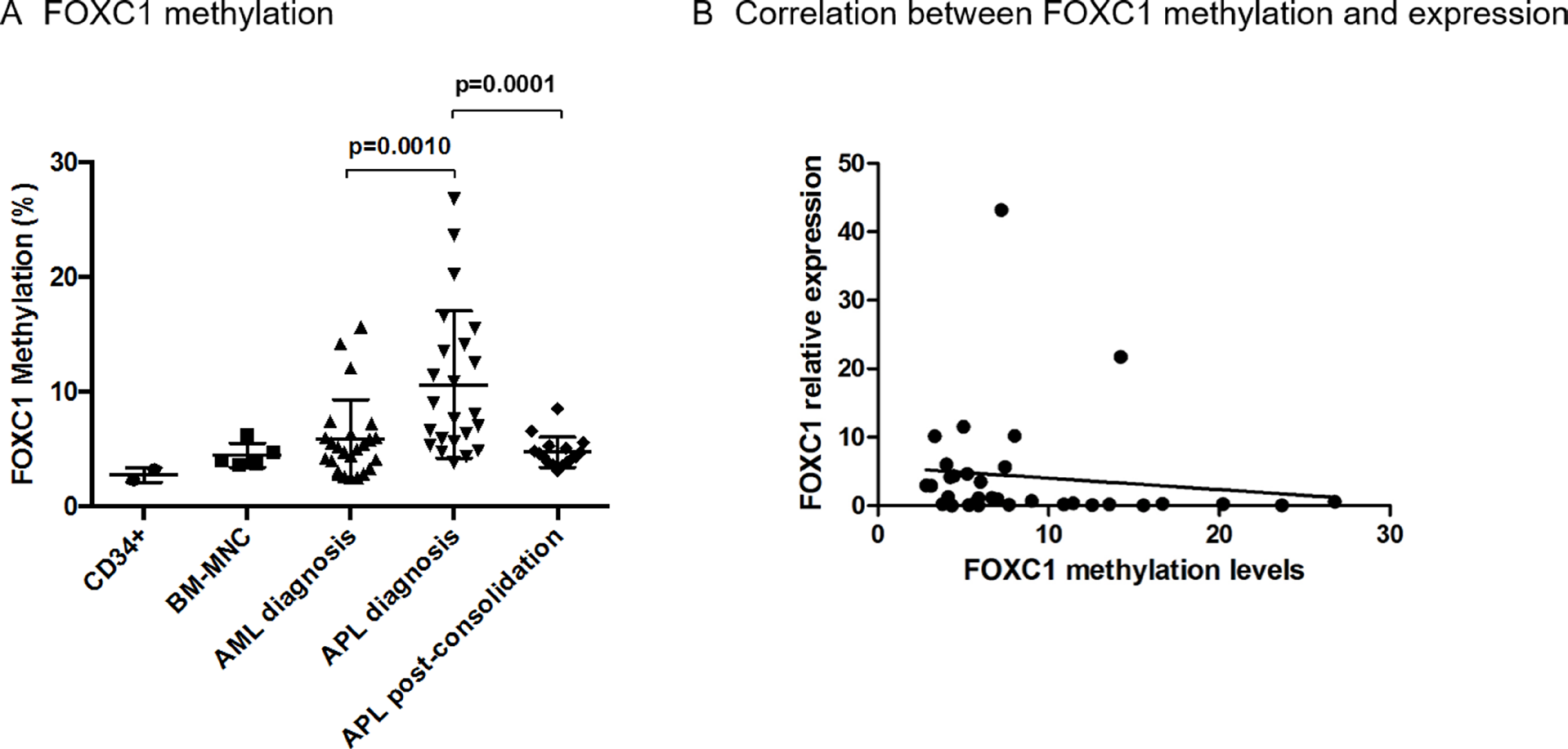
FOXC1 methylation levels (**A**) FOXC1 methylation levels in DNA extracted from cord blood CD34+ samples (*n* = 2), BM-MNC samples from healthy donors (*n* = 5), AML and APL patients at initial diagnosis (*n* = 24 and *n* = 23 samples, respectively), and from APL patients after consolidation treatment (*n* = 16). (**B**) Correlation between FOXC1 methylation and mRNA expression in individual patients with APL (*n* = 19) and AML (*n* = 14).

Since FOXC1 expression has been shown to be regulated by DNA methylation [19], we quantitatively analysed FOXC1 methylation in the DNA region spanning bp +354 to +568 from the TSS, using a specific pyrosequencing assay. Methylation of FOXC1 was significantly higher in APL samples compared to non-APL AML (mean methylation 11% in 23 APL, vs 6% in 24 AML *p* = 0.010, Figure 2A), and decreased after consolidation treatment in all APL tested (16 APL, mean 5%, *p* = 0.0001, Figure 2A). In these APL samples collected at the time of complete remission, methylation levels were similar to that of cord blood CD34+ cells and BM-MNC samples collected from healthy donors (2 CD34+ and 5 BM-MNC samples), as well as to that of AML samples at diagnosis (Figure 2A). A significant inverse correlation between FOXC1 expression and methylation levels was confirmed in 19 APL and 14 AML samples with available data (R= –0.35, *p* = 0.05, Figure 2B). Overall, our data show that FOXC1 expression is downregulated by DNA hypermethylation in APL.

### Modulation of FOXC1 expression during the process of normal granulocytic differentiation

**Figure 3:**
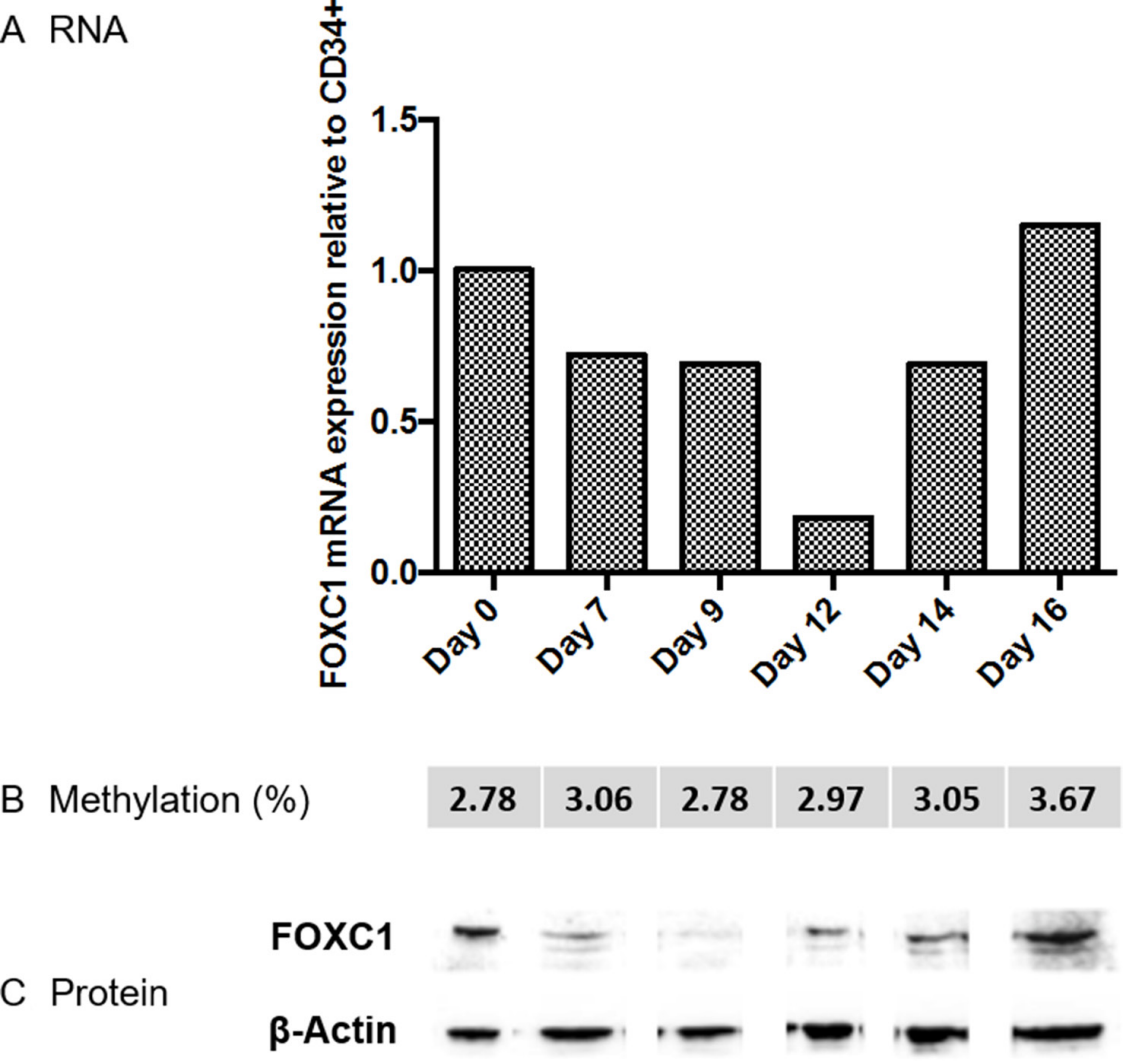
FOXC1 expression during normal granulocytic differentiation FOXC1 mRNA and protein relative expression and methylation level (indicated as average percent methylation of 9 CpG sites (met %)) in cord blood CD34+ cell samples, at time 0 and after differentiation along the granulocytic lineage. Specific time points during granulocytic differentiation are indicated in the graph. Western blot images are assembled for figure usability in respect of original image acquisition and no specific features are obscured, moved, removed, or introduced.

Since APL is characterized by a maturation block at the promyelocyte stage, removed by ATRA, we investigated the kinetics of FOXC1 expression during the early phases of normal granulocytic differentiation, using an *in vitro* differentiation model starting from CD34+ cells. FOXC1 expression studied by quantitative RT-PCR initially decreased, and subsequently increased on day 16. Western blot analysis of FOXC1 protein expression confirmed this pattern. Different from APL, FOXC1 downregulation during the early phases of granulocytic differentiation was not associated to changes in FOXC1 methylation levels (Figure 3). On the other hand, the kinetics of FOXC1 expression during *in vitro* differentiation along the erythroid, megakaryocytic and monocytic lineages were characterized by a decrease of FOXC1 mRNA expression levels (Supplementary Figure 3).

### Characterization of FOXC1 expression in leukemic cell lines

We then used the APL leukemic cell line NB4, the myeloblastic and monoblastic cell lines HL60 and PR9 (derivative of U937, with zinc-inducible PML/RARA), and the non-hematopoietic cell line HEK, to characterize the regulation of FOXC1 expression. In agreement with the observations made using primary APL blasts, we detected lower FOXC1 mRNA levels in NB4, HL60 cells (PML/RARA-negative) and PR9 cells, while HEK cells displayed markedly higher FOXC1 expression (Supplementary Figure 2A). Similar differences were observed at the protein level. Correspondingly, FOXC1 methylation levels were lower in HEK cells (2%), than in NB4, HL60 and U937 cells (61%, 48%, and 48%, respectively, Supplementary Figure 2B).

### Modulation of FOXC1 expression by PML-RARA

**Figure 4:**
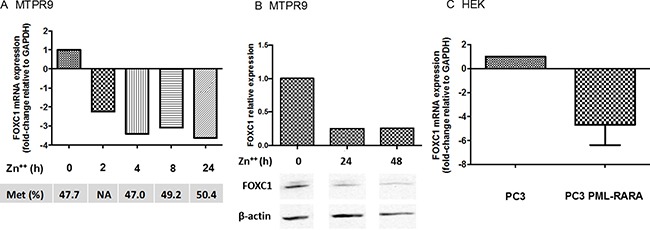
Expression of FOXC1 in cell lines transfected with PML-RARA In MTPR9 cells, a monoblastic cell line (U937) expressing PML-RARA under the control of a Zn^2+^ -inducible promoter, FOXC1 mRNA (**A**) and protein (**B**) expression were evaluated after transfection of PML-RARA constructs (western blot images are assembled for figure usability in respect of original image acquisition and no specific features are obscured, moved, removed, or introduced). Corresponding FOXC1 methylation status is also shown. FOXC1 mRNA expression was also studied in embryonic kidney (HEK) cells transfected with an empty pSG5 vector or with a pSG5 vector containing the PML-RARA fusion gene (**C**).

We then explored the effects of the PML-RARA fusion gene on FOXC1 expression. In the PR9 cell line, induction of PML-RARA by addition of Zn^2+^ down-modulated the level of FOXC1 mRNA and protein expression (Figure 4A and 4B). However, pyrosequencing assays showed that FOXC1 methylation status did not significantly change following induction of PML/RARA expression.

To further explore the effects of PML/RARA on FOXC1, we transfected the human HEK kidney cell line with a pSG5 vector containing the PML-RARA gene, or with an empty pSG5 vector as a negative control. Again in these cells, transfection of PML-RARA markedly decreased FOXC1 levels compared to the control (Figure 4C). Similar to PR9 cells, transfection of PML-RARA did not significantly impact on FOXC1 methylation.

**Figure 5:**
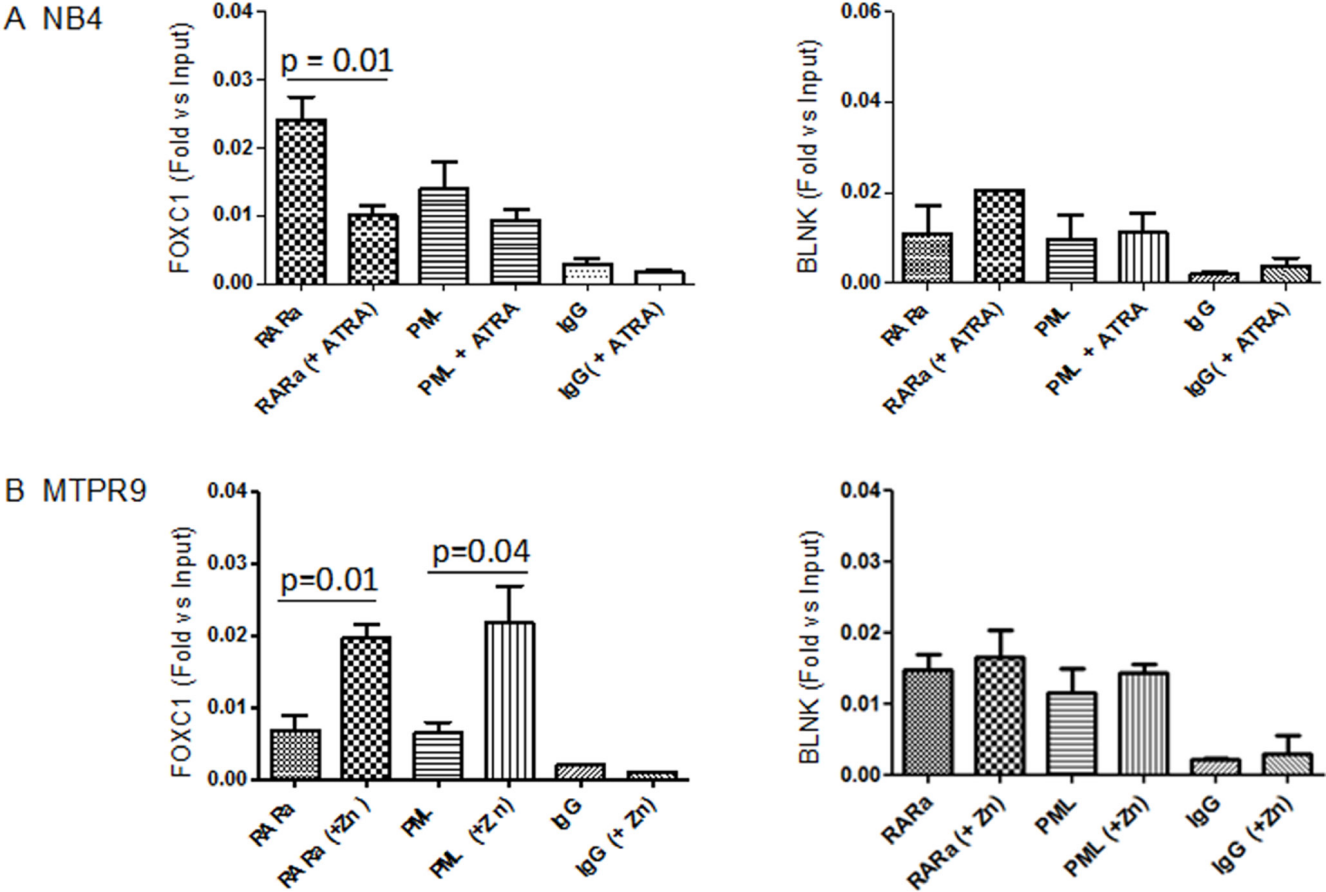
Binding of PML-RARA to the FOXC1 promoter region (**A**) PML and RARA binding to FOXC1 DNA, as measured in NB4 cells by a quantitative ChIP assay, followed by amplification of the FOXC1 promoter region by RT-qPCR, before and after treatment with ATRA. A rabbit polyclonal IgG antibody was used as control. (**B**) PML-RARA binding to FOXC1 DNA in PR9 cells treated with Zn(SO_4_), which induces PML-RARA overexpression. Data are shown as fold enrichment of ChIP DNA versus input DNA, and are representative of at least three independent experiments. The promoter region of the BLNK gene was used as negative control. The *p*-value was calculated by the unpaired *t*-test.

We then tested the binding of PML-RARA to the promoter region of FOXC1 using the NB4 cell line, and anti-PML or anti-RARA antibodies. PML-RARA bound to motif –398 to –391 of the FOXC1 promoter and negatively regulated its expression. In this context, addition of ATRA was associated to a decrease of PML-RARA binding to the FOXC1 promoter (Figure 5A). These data were confirmed in PR9 cells, where induction of PML-RARA expression by addition of Zn++ increased the binding of PML-RARA to the FOXC1 promoter region (Figure 5B). These results indicate that differentiation of APL induced by ATRA treatment unlocks FOXC1 promoter, inducing upregulation of its expression.

### Pharmacological modulation of FOXC1 expression *in vitro*

**Figure 6:**
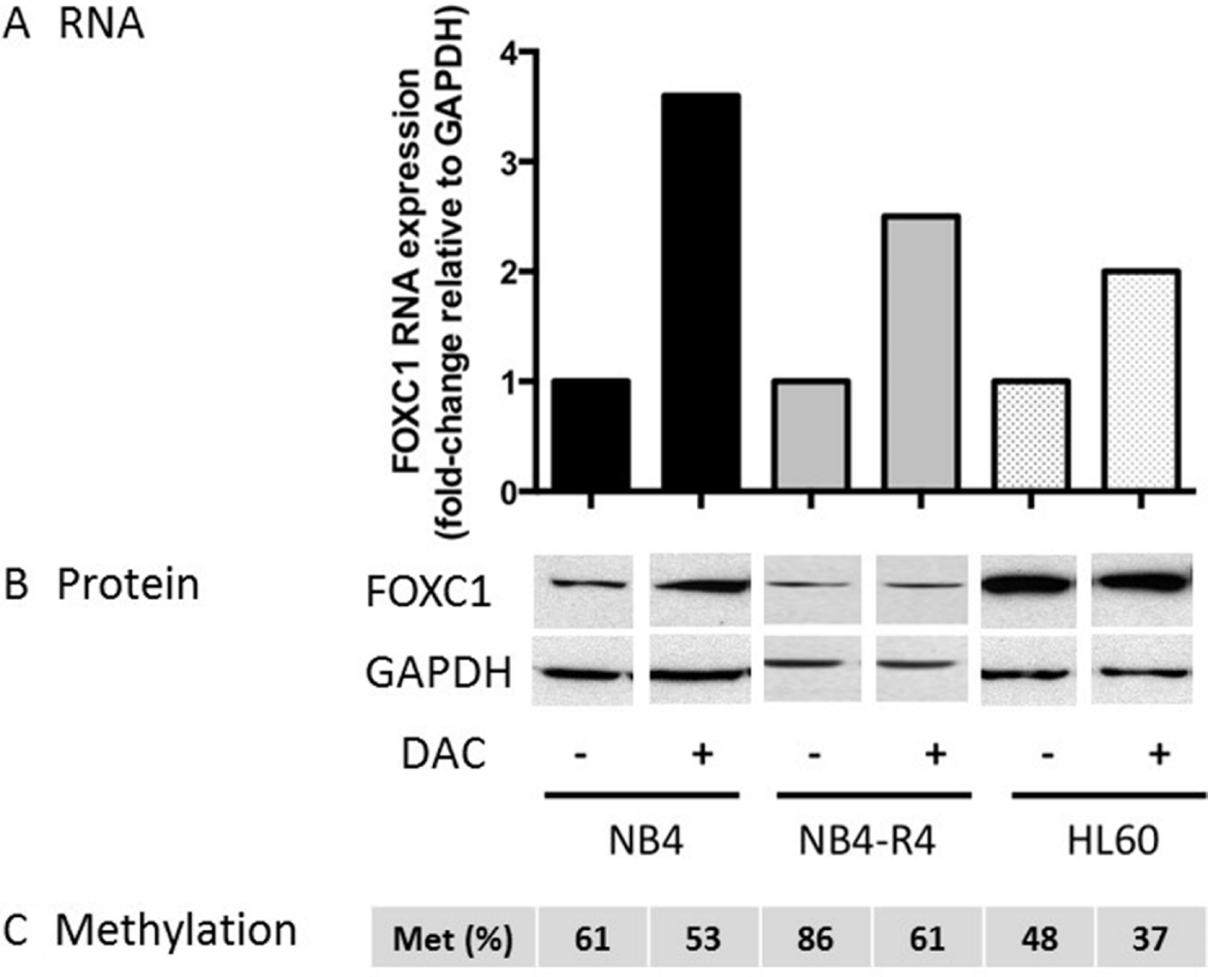
FOXC1 expression after decitabine treatment (**A**) FOXC1 mRNA and protein (**B**) expression in ATRA-treated versus untreated cells. GAPDH was used as control for protein quantification (three different western blot images, NB4 treated and untreated, NB4-R4 treated and untreated and HL60 treated and untreated cells, are assembled for figure usability in respect of original image acquisition and no specific features are obscured, moved, removed, or introduced). (**C**) FOXC1 DNA methylation level (%).

Treatment of the NB4 and HL60 cell lines with the hypomethylating agent decitabine (DAC 1 μM), induced a moderate decrease of FOXC1 methylation compared to baseline (mean 53% and 37% in DAC-treated vs 61% and 37% in NB4 and HL60 control cells, respectively, Figure 6C). This was associated to upregulation of FOXC1 mRNA expression (fold-change 3.6, *p* = 0.04 and 2.0, *p* = 0.05 in NB4 and HL60 cells, respectively, Figure 6A). FOXC1 protein levels were only slightly upregulated (Figure 6B). Altogether, our data show that FOXC1 regulation by methylation is functional in hematopoietic cell lines, similarly to primary APL samples.

**Figure 7:**
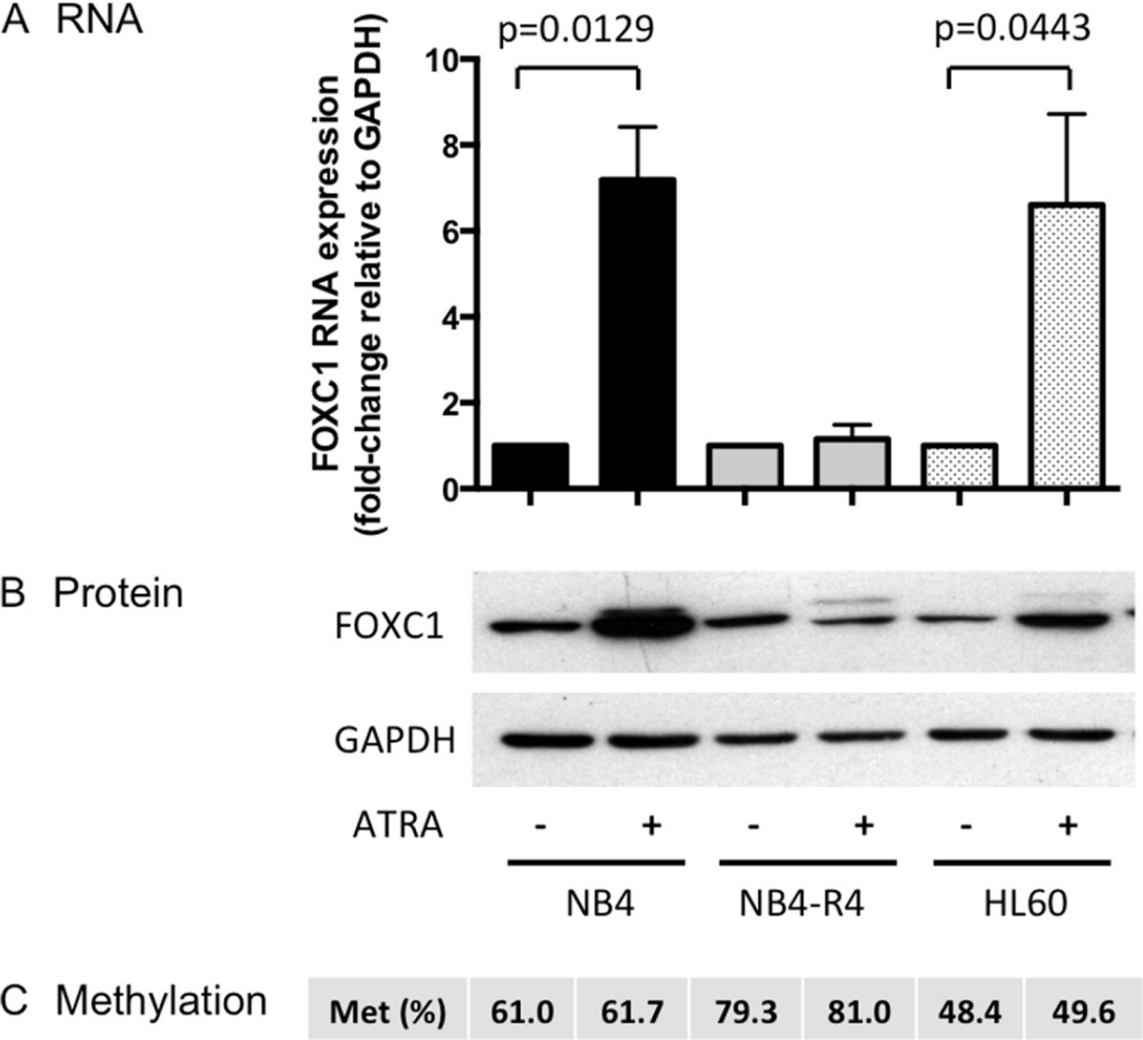
FOXC1 expression after ATRA treatment (**A**) FOXC1 mRNA and protein (**B**) expression in ATRA-treated versus untreated cells. GAPDH was used as control for protein quantification (western blot images are assembled for figure usability in respect of original image acquisition and no specific features are obscured, moved, removed, or introduced). (**C**) FOXC1 DNA methylation level (%).

Granulocytic differentiation can be induced in NB4 and HL-60 cell lines by ATRA. We then studied the regulation of FOXC1 expression after ATRA treatment (Figure 7). ATRA induced marked upregulation of FOXC1 both at mRNA (fold-change 7.2, *p* = 0.0129, and 6.6, *p* = 0.0443, in NB4 and HL60 cells, respectively, Figure 7A) and protein levels (Figure 7B). However, this was not associated with changes in FOXC1 methylation (Figure 7C), indicating a methylation-independent mechanism in ATRA-treated NB4 and HL-60 cells.

The combination of ATRA and decitabine treatment was not synergic on induction of FOXC1 mRNA expression, compared to either agents alone (Supplementary Figure 4). Also, treatment with arsenic trioxide, which is a very active drug in patients with APL, did not affect FOXC1 expression in NB4 cells (Supplementary Figure 5).

Of note, upregulation of FOXC1 did not occur upon ATRA treatment of ATRA-resistant NB4-R4 cells [20] (Figure 7A–7C), whereas decitabine treatment was able to induce FOXC1 demethylation and mRNA up-regulation also in this cell line (fold-change 2.5, *p* = 0.04, Figure 6A–6C). These data show that ATRA regulates FOXC1 expression through the RA receptor and decitabine treatment may restore FOXC1 expression in ATRA-resistant APL cells.

## DISCUSSION

We show here that FOXC1 mRNA and protein expression are significantly downregulated in APL, compared to other AML subtypes. Our findings were confirmed by interrogating the TCGA data sets, where APL displayed 10-fold lower FOXC1 expression than the majority of non-APL AML. Previous studies reported functional overexpression of FOXC1 in about 20% of primary AML samples [13], but a specific analysis on APL cases had not been reported so far.

Interestingly, FOXC1 expression in the majority of AML was similar to that observed in normal hematopoietic progenitors and mature myeloid cells. Our data on CD34+ cells differ from those of Somerville *et al,* who reported absence of FOXC1 in murine BM progenitor cells [13]. Using a semi-quantitative PCR assay developed by Omatsu *et al.* [15], we found that FOXC1 mRNA expression in normal CD34+ progenitor cells was low, but clearly detectable at both the mRNA and protein level. Following *in vitro* differentiation along the granulocytic lineage, FOXC1 expression was initially downregulated and subsequently restored through a methylation-independent mechanism.

The observed FOXC1 downregulation in APL was however at least in part due to aberrant DNA hypermethylation, identified in CpGs localized next to the TSS (from + 354 to + 368), whose regulatory role has been previously shown by Klajic *et al.* in breast cancer cells [21]. Our observations are in apparent contrast with the lack of chromatin compaction and FOXC1 methylation in hematopoietic cells shown by Somerville *et al.* [13], who indeed analyzed a region located in the FOXC1 promoter. Epigenetic regulation of FOXC1 expression in non-hematopoietic cells was also confirmed in a study conducted by Yang *et al.*, in a large cohort of patients with idiopathic pulmonary fibrosis. These authors showed that a single differentially methylated region (DMR) localized close to the FOXC1 TSS was able to drive changes in its expression [19]. Likewise, Zhang *et al.*, recently identified functional DNA methylation changes in midbrain dopaminergic neurons of patients affected by Parkinson disease [20].

We further confirmed the epigenetic modulation of FOXC1 expression using hypomethylating agents. In fact, treatment of NB4 and HL60 cell lines with decitabine, induced a decrease of FOXC1 methylation and a concomitant upregulation of its expression. Similar results were recently reported by Wang *et al.*, in melanoma cell lines, using the hypomethylating agent azacitidine [12]. These investigators found that FOXC1 overexpression was associated with disease progression and poor prognosis, and suggested FOXC1 as a potential prognostic biomarker to predict outcome [12].

Using *in vitro* models mimicking the process of normal granulocytic differentiation, we found that FOXC1 expression was initially downmodulated, and subsequently upregulated, independently of DNA methylation. In NB4 and HL-60 leukemic cell lines, differentiation along the granulocytic lineage using ATRA was associated to marked FOXC1 upregulation, without any changes in DNA methylation, suggesting the contribution of an ATRA-dependent mechanism to FOXC1 modulation. Using an ATRA-resistant APL cell line [22], we confirmed that FOXC1 binds to the RARA receptor.

Following these observations, we explored the effects of the PML-RARA fusion gene on FOXC1 expression using the PR9 inducible cell line. Induction of PML-RARA expression down-regulated FOXC1 expression, clearly showing the pivotal role of this fusion gene in FOXC1 regulation in APL cells and in non-hematopoietic cell line. We then showed that FOXC1 downregulation in APL is due to binding of PML/RARA to a specific motif localized in the FOXC1 promoter region (from –398 to –391), and that ATRA addition decreases PML-RARA binding and unlocks FOXC1 in both NB4 and PR9 cell lines. This mechanism may, at list in part, explain the silencing of FOXC1 expression in APL samples and the restoration of its expression *in vivo* after ATRA/ATO or ATRA and chemotherapy treatment. The lack of synergism between ATRA and decitabine *in vitro* may be due to the primary differentiating effect of ATRA in APL, which may limit the incorporation of decitabine in the DNA.

In conclusion, our data show that down-modulation of the transcription factor FOXC1 is a consistent feature of APL, occurring as a consequence of binding of the oncogenic protein PML-RARA to the FOXC1 promoter, and to FOXC1 hypermethylation. Further studies in mice models are needed to explore the pathogenetic and therapeutic implications of these observations. The potential role of hypomethylating agents in advanced APL, alone or in combination with other agents (e.g. ATRA and/or ATO) remains to be established.

## MATERIALS AND METHODS

### Cell line culture and *in vitro* treatment

NB4, an APL-derived t(15;17)-positive cell line, was purchased from DSMZ (Braunschweig, Germany). The ATRA-resistant cell line, NB4-R4, was kindly donated by Clara Nervi (La Sapienza University, Rome, Italy). The myeloid cell lines HL60, U937, and PR9, a zinc-inducible PML/RARA model derived from U937 cells, were kindly provided by Emanuela Colombo (European Institute of Oncology, Milan, Italy). Hek293T (HEK), a human embryonic kidney cell line, was kindly provided by Corinna Giorgi (European Brain Research Institute, EBRI, Rome, Italy).

All cell lines, but HEK, were grown at 37°C in a humidified atmosphere of 5% CO_2_ in air, in RPMI medium (GIBCO-BRL, Grand Island, NY, USA), supplemented with 10% fetal bovine serum (FBS) (GIBCO-BRL), 20 mM Hepes, 100 U/mL penicillin and 100 μg/mL streptomycin (GIBCO-BRL). HEK cells were grown in DMEM (GIBCO-BRL, Grand Island, NY, USA).

All-trans retinoic acids (ATRA), decitabine, ZnSO_4_ and Arsenic trioxide (all from Sigma-Aldrich, Steinheim, Germany), were dissolved in DMSO (10 mM stock solution, SS), Acetic Acid 50% (10mM SS), water (1M SS) and PBS (1x), respectively. DAC was added to the culture medium at 1 μM for 3 days, whereas ATO and ATRA were added to the culture medium at 1 μM for 1 day, as previously reported [3, 23]. ZnSO_4_ was added to the culture medium at 100 μM for 1 day [3]. Acetic Acid 50% and DMSO were used as control.

### Patient and controls

The study population included 54 patients (29 females and 25 males, median age: 48.7 years, range: 14–87 years), with newly diagnosed AML (27 APL and 27 consecutively admitted other AML subtypes), whose BM samples were collected at the time of initial diagnosis. In 11 of the 27 patients with APL, BM samples were also studied at the time of remission following consolidation treatment using ATRA and chemotherapy or ATRA/arsenic trioxide or ATRA alone (*n* = 19, *n* = 7 and *n* = 1, respectively), according to the AIDA2000 and APL0406 studies [17–18]. The diagnosis was established according to standard morphologic, immunophenotypic and genetic criteria, according to the World Health Organization (WHO) classification [24]. Main patients demographic and clinical features are detailed in Table 1.

**Table 1:** Patient characteristics

	AML (*n*: 27)	APL (*n*: 27)
**Age (median, range)**	56 (15–87)	41 (14–82)
**Sex (F/M)**	14/13	15/12
**Karyotype (*n*** **= 18)**	7	na
**Normal**	2	
**t(8;21)(q22;q22.1)**	4	
**inv(16)(p13.1q22) or t(16;16)(p13.1;q22) Complex**	4	
**Hyperdiploid**	1	
**Molecular genetics**		
FLT3-ITD	6	
NMPM1-mut	3	
**PML/RARA breakpoint**		
**Bcr1**		18
**Bcr2**		2
**Bcr3**		7
**Hb (g/dl)**	9.4	9.5
**WBC 10^9^/L (median, range)**	29.8 (0.4–122)	6.7 (5.3–45)
**PLTS 10^9^/L (median, range)**	43 (5.5–79)	53 (0.4–180)
**Sanz Risk score** [16]		
**Low/intermediate**		24
**High**		3

na: not available

Mononuclear cells (MNC) were obtained by Ficoll gradient centrifugation using Lympholyte-H (Cedarlane, Ontario, Canada). Hematopoietic CD34+ progenitor cells isolated from 2 cord blood (CB) obtained from healthy full-term placentas, and 5 BM-MNC harvested from healthy donors, monocytes (*n* = 9), or neutrophils (*n* = 3), isolated from peripheral blood (PB), were used as controls. CD34+ cells were purified from CB and BM by positive selection using the midi-MACS immunomagnetic separation system (Miltenyi Biotec, Bergisch Gladabach, Germany), according to the manufacturer's instructions. The purity of CD34+ cells was assessed by flow cytometry using a monoclonal PE-conjugated anti-CD34 antibody and resulted over 95% (range 92–98%). Purified human hematopoietic progenitor cells were grown in serum-free medium containing BSA (10 mg/ml), pure human transferrin (1 mg/ml), human low-density lipoproteins (40 μg/ml), insulin (10 μg/ml), sodium pyruvate (10^–4^ M), L-glutamine (2 × 10^–3^ M), rare inorganic elements (Sn, Ni, Va, Mo and Mn) supplemented with iron sulphate (4 × 10^–8^ M) and nucleosides (10 μg/ml each). CD34+ cells were induced into granulocytic differentiation by addition of IL-3 (1 unit/ml), granulocyte/monocyte CSF (0.1 ng/ml) and saturating amounts of G-CSF (500 units/ml); megakaryocytic differentiation with Thrombopoietin (TPO) (50 ng/ml); monocytic differentiation with M-CSF (10 ng/ml), FLT3 Ligand (50 ng/ml) and IL-6 (10 ng/ml); erythroid differentiation with erythropoietin (Epo) 3U/ml, IL-3 (0.01 unit/ml) and GM-CSF (0.01 ng/ml). The differentiation stage was evaluated by May Grunwald-Giemsa staining (Sigma-Aldrich, St. Louis, Mo, USA) and cytologic analysis (data not shown).

The study was approved by the Ethics committee of Tor Vergata University (Rome, Italy), and informed consent was obtained from all patients and controls, according to the Declaration of Helsinki.

### Nucleic acid isolation and reverse transcription qPCR

DNA was extracted using the QIAamp DNA Mini Kit (Qiagen AG, Milan, Italy), following the manufacturer's instructions. Total RNA was extracted using the RNeasy Mini Kit (Qiagen AG, Milan, Italy). Complementary DNA used for reverse transcription quantitative PCR (RT-qPCR) was synthesized using the QuantiTect Reverse Transcription Kit (Qiagen AG, Milan, Italy), in accordance with the manufacturer's instructions. Expression levels of FOXC1 mRNA were analysed through a semi-quantitative PCR assay [15] (iQ SYBR Green Supermix, Bio-Rad), using GAPDH as reference gene. A melting curve (62°C–95°C) was generated at the end of each run to verify specificity of the reactions. Specific gene expression values, used to compare APL and AML samples, were expressed as 2^–ΔCt^, where ΔCt = Ct (test gene) - Ct (reference gene). Specific gene expression values, used to compare *in vitro* treatment, were expressed as 2^–ΔΔCt^, where ΔΔCt = ΔCt (test gene) - ΔCt (reference gene).

### FOXC1 methylation

Bisulfite treatment and specific-pyrosequencing assays were performed to analyze the methylation status of FOXC1. Briefly, genomic DNA (1 μg) was bisulfite-converted using the EZ DNA Methylation-Gold Kit^TM^ (Zymo Research, Freiburg, Germany). Specific primers and annealing conditions have been previously reported by Muggerud *et al.* [25] The target region spans from +354 to +568 (nt 65513-65727, EMBL: AL034344) from the transcription start site (TSS). Reagents and protocols used for quantitative DNA methylation analysis were as recommended by manufacturers (PyroMark Q96 ID, Diatech Pharmacogenetics, Jesi, Italy).

### Western blot analysis

Cell pellets were re-suspended in lysis buffer containing 10 mM Tris-HCl (pH 7.4), 5 mM EDTA, 150 mM NaCl, 1% Triton X-100, 250 μM orthovanadate, 20 mM β-glycerophosphate and protease inhibitors (Sigma-Aldrich, Steinheim, Germany). Lysates were centrifuged at 10000 g for 15 minutes at 4°C and supernatants were stored at −80°C. Protein concentration was measured by the Bradford Assay (#500––0006; Bio-Rad, München, Germany). Thirty microgram aliquots of proteins were re-suspended in a reducing Laemmli Buffer (with β-mercaptoethanol), loaded onto a 10% polyacrylamide gel, and then transferred to nitrocellulose membrane. After blocking with 5% milk (Fluka, Sigma-Aldrich, Saint Louis, USA), the membranes were incubated with anti-FOXC1 antibody code 8758S, cloneD8A6 (Cell Signalling Technology, Beverley, MA, USA). Horseradish peroxidase-conjugated IgG preparations were used as secondary antibodies, and the immunoreactivity was determined by the enhanced chemiluminescence (ECL) method (Amersham, Buckinghamshire, UK). The autoradiograms were scanned and exported for densitometry analysis. Protein signal intensities were measured using the Quantity One Software (Bio-Rad Laboratories, Hercules, CA, USA). Signal quantity was normalized using the unrelated proteins β-actin (Cell Signaling Technology, Beverley, MA, USA).

### ChIP assay

ChIP assays were performed as previously described by Simone *et al.*, with some modifications [26]. Briefly, DNA was double-crosslinked to proteins with 1% formaldehyde (Sigma, St Louis, USA). After incubation for 10 minutes at room temperature, glycine was added to a final concentration of 0.125 M, for 5 minutes. The cells were washed twice with PBS 1×, cell lysis buffer (10 mM Tris pH 8.0, 100 mM NaCl and 0.2% NP40) was then added to the samples and incubated on ice for 30 minutes. Nuclei were pelleted by microfuge at 1500 RPM at 4°C, and after addition of the nuclear lysis buffer (50 mM Tris 8.1, 10 mM EDTA and 1% SDS) were incubated on ice for 30 minutes. Chromatin fragments of around 200–300 bp were obtained by sonication, using a Branson Sonifier 450 Analog Cell Disruptor (30″ ON, 45″ OFF, for a total time of 10 minutes at output 2). For each immunoprecipitation, 2 mg of antibodies were conjugated to magnetic beads (G-protein magnetic Beads, Invitrogen, Dynal, Oslo). The following antibodies were used in the ChIP assays: anti-RARA (C-20, sc-551X, Santa Cruz Biotechnology, Inc. Dallas, USA), anti PML (H-238, sc5621, Santa Cruz Biotechnology, Inc. Dallas, USA), and a rabbit polyclonal IgG (#2729, Cell Signaling Inc. Massachusetts, USA). After extensive washing, bound DNA fragments were eluted and analyzed by quantitative PCR using the SYBR Green Master Mix (Applied Biosystems, Warrington, UK). ChIP signals were normalized against the input and expressed as relative enrichment of the material, precipitated by the indicated antibody binding to the FOXC1 promoter [relative quantification using the comparative Ct method (2−(Ct sample−Ct input)]. Chip primers were: CHIP-Foxc1-F: 5′-CGCCTGCTTGTTCTTTCTTT-3′, CHIP-Foxc1-R: 5′-CCGCCTTGCAGGAACTC-3′. The BLNK gene was used as a negative control; BLNK-F: 5′- GGCCCTGACTGATGGAAATTAC -3′ and BLNK-R: 5′- CAGCAGGTGACCATCCCTTTAG -3′.

### Analysis of TCGA data

Gene expression analysis data from AML samples deriving from the The Cancer Genome Atlas Research Network 2008 were directly obtained from the public access data portal (http://cancergenome.nih.gov/dataportal/data/about). In this analysis, 179 AML samples stratified by the French American British morphology classification (FAB) and a total of 20319 genes with expression values in the RPKM format were included. Expression of FOXC1 in the 179 AML samples was plotted according to 5 FAB categories (Supplementary Figure 1). Data sets were cross-referenced using tumor-specific identification numbers.

### Statistical analysis

The GraphPad Prism Statistical PC program (GraphPad Software, San Diego, CA) was used for statistical analysis. Data are presented as mean ± SD or median and range. Grouped data were compared using the non-parametric Mann–Whitney *U* test. *p*-values ≤ 0.05 were considered statistically significant. Prediction of transcription factor binding sites was performed using the LASAGNA-Search 2.0 Software (http://biogrid.engr.uconn.edu/lasagna_search/).

## SUPPLEMENTARY MATERIALS FIGURES


