# Supplementary material for: The forkhead box C1 (FOXC1) transcription factor is downregulated in acute promyelocytic leukemia
Source: Oncotarget. 2017 Sep 20;8(48):84074–85. doi: 10.18632/oncotarget.21101 (PMC5663578; doi:10.18632/oncotarget.21101)
Supplement: Supplementary file 1 [file oncotarget-08-84074-s001.pdf]

The forkhead box C1 (FOXC1) transcription factor is downregulated in acute promyelocytic leukemia

SUPPLEMENTARY MATERIALS

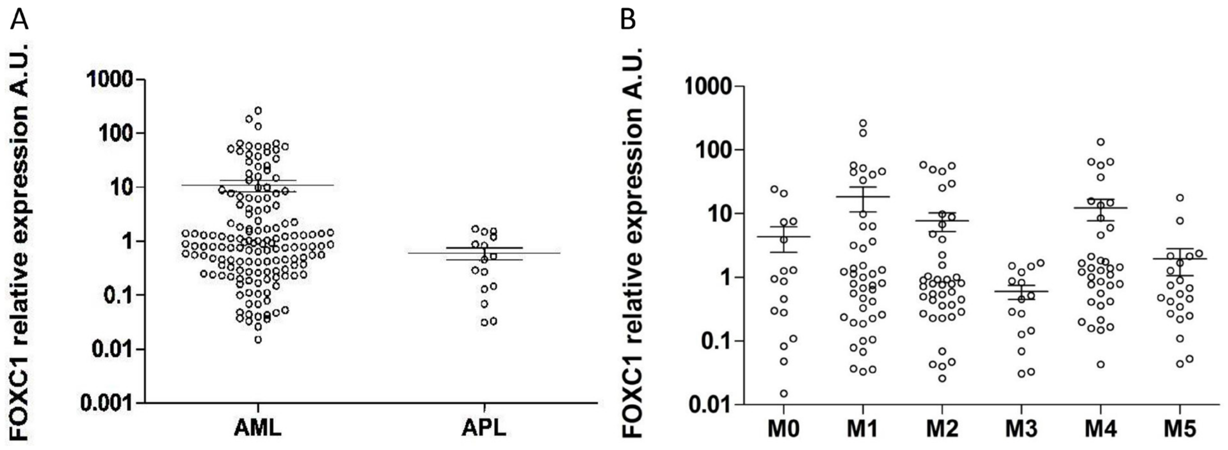

Supplementary Figure 1: (**A**) Data derived from the TGCA data set, indicating that FOXC1 mRNA expression is lower in APL compared to other AML subtypes. (**B**) Detailed FOXC1 mRNA gene expression in AML samples, grouped according to the FAB classification.

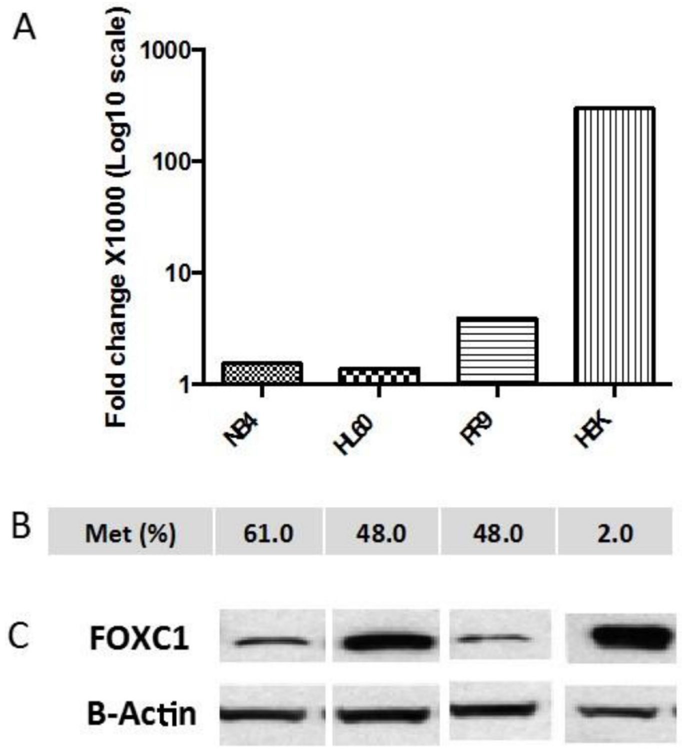

Supplementary Figure 2: FOXC1 mRNA relative expression, methylation (%) and protein levels in the NB4 (PML/RARA+) and HL60 (PML/RARA–) promyelocytic cell lines, the PR9 cells, a derivative of U937 monoblastic cell line and a non-hematopoietic cell line (HEK:human embryonic kidney 293). (**A**) FOXC1 mRNA relative expression (Log10 scale). (**B**) FOXC1 methylation level (%) (**C**) FOXC1 protein expression relative to β-Actin.

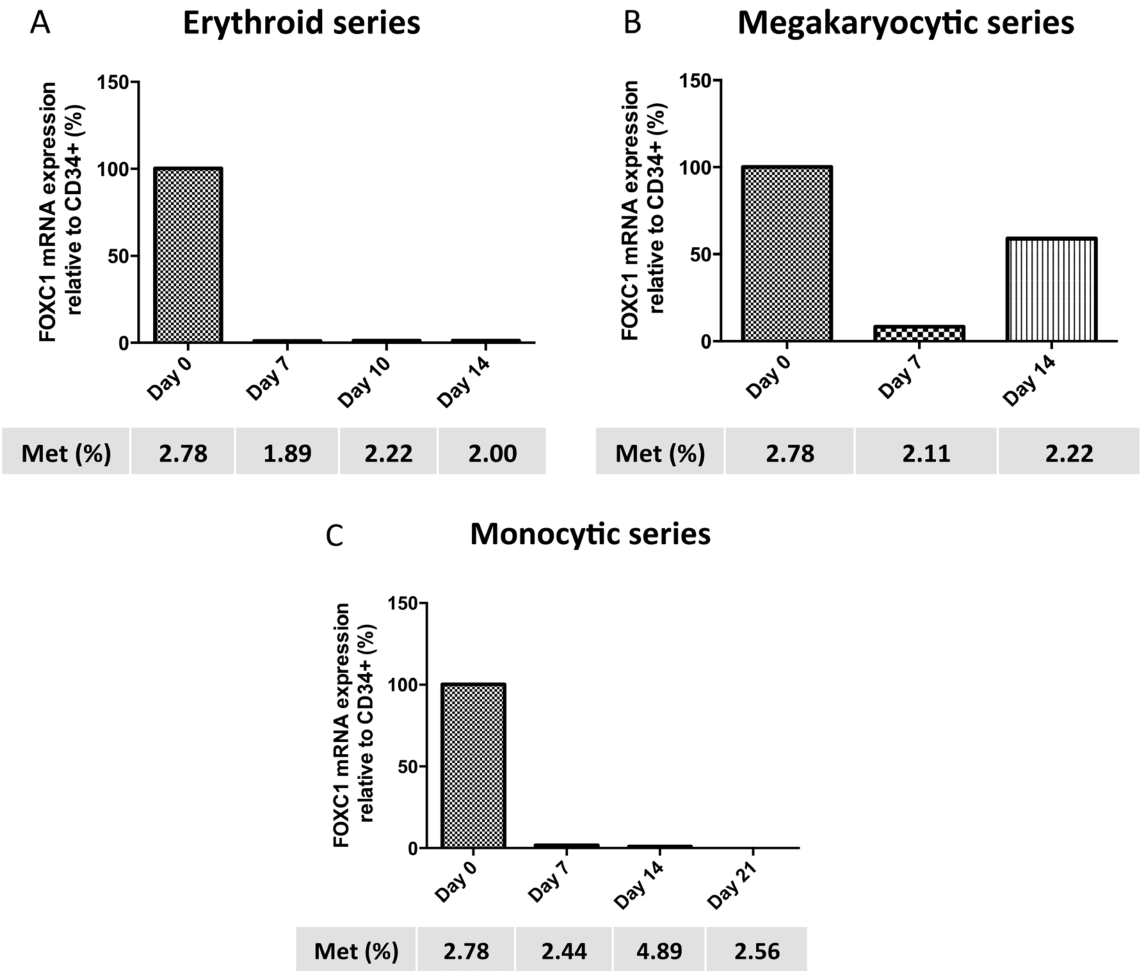

Supplementary Figure 3: FOXC1 mRNA relative expression and methylation level (indicated as average percent methylation of 9 CpG sites (met %)) in cord blood CD34+ cell samples, at time 0 and after differentiation along the different lineages (**A)** erythroid; (**B**) megakaryocytic; (**C**) monocytic. Differentiation time points are indicated in the graph.

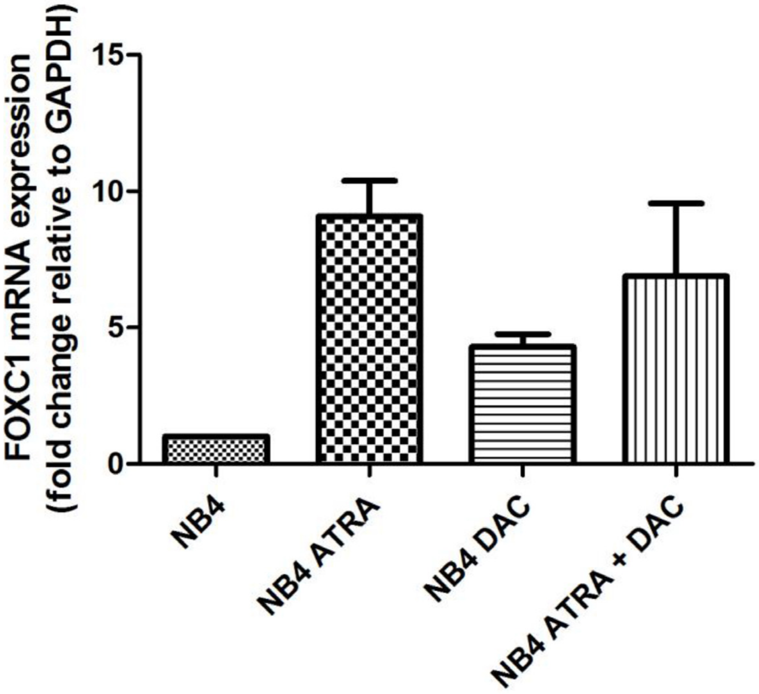

Supplementary Figure 4: FOXC1 mRNA relative expression in NB4 cells, and after treatment with ATRA, decitabine (DAC), and the ATRA-DAC combination.

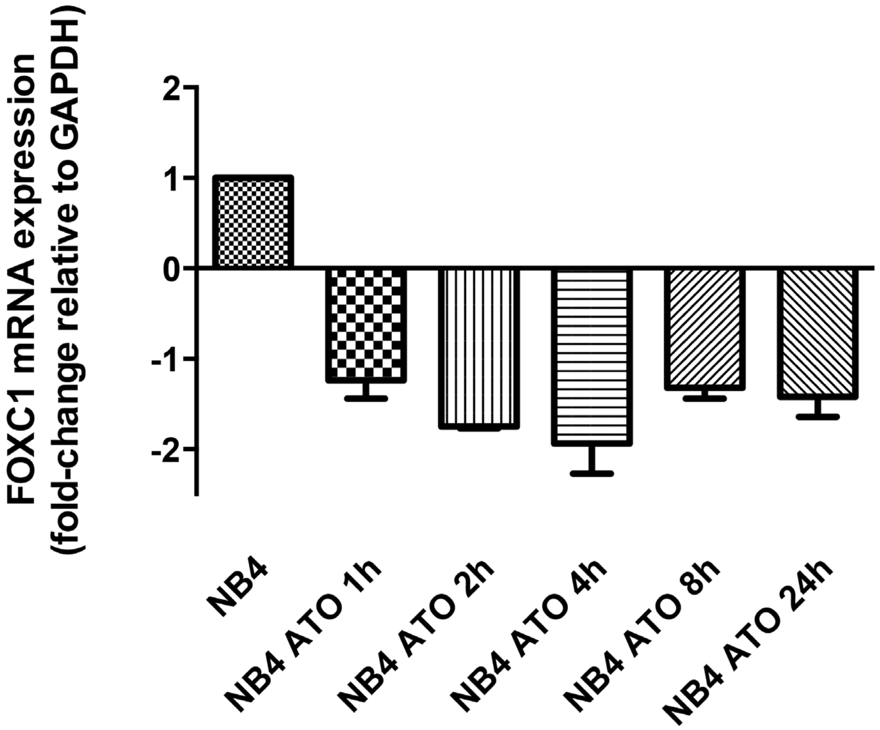

Supplementary Figure 5: FOXC1 mRNA relative expression in NB4 cells, and after treatment with ATO (1 hour to 24 hour time points).
